# Brief Review of Right Aortic Arch with Aberrant Left Subclavian Artery

**DOI:** 10.1055/s-0039-3401999

**Published:** 2020-02-17

**Authors:** Didem Melis Oztas, Muzaffer Umutlu, Melike Ertan, Metin Onur Beyaz, Serdar Badem, Ibrahim Erdinc, Mustafa Ozer Ulukan, Orcun Unal, Cenk Conkbayir, Ufuk Alpagut, Murat Ugurlucan

**Affiliations:** 1 Department of Cardiovascular Surgery, Bagcilar Training and Research Hospital, Istanbul, Turkey; 2 Department of Radiology, Istanbul University Istanbul Medical Faculty, Istanbul, Turkey; 3 Department of Cardiovascular Surgery, Istanbul University Istanbul Medical Faculty, Istanbul, Turkey; 4 Department of Cardiovascular Surgery, Istanbul Medipol University Medical Faculty, Istanbul, Turkey; 5 Department of Cardiovascular Surgery, Bursa City Hospital, Bursa, Turkey; 6 Department of Cardiovascular Surgery, Izmir Bozyaka Training and Research Hospital, Izmir, Turkey; 7 Department of Cardiovascular Surgery, Yedikule Chest Diseases and Thoracic Surgery Training and Research Hospital, Istanbul, Turkey; 8 Department of Cardiology, Near East University, Nicosia, Cyprus

**Keywords:** subclavian artery, aberrant, aortic arch

## Abstract

Development anomalies of the aortic arch and its major branches are rare congenital cardiovascular disorders. In this article, we present aberrant left subclavian artery associated with right aortic arch.

Right aortic arch (RAA) is a rare malformation and is reported at a range of 0.04 to 0.1% in autopsy series. 1 The anomaly occurs in embryonic life due to the persistence of the right-fourth aortic arch, while regressing the left-fourth arch between the left common carotid artery and the left subclavian artery. 2 There are three types of the right-sided aortic arch as follows: Type I involves right aortic arch with mirror image branching, Type II involves right aortic arch with aberrant left subclavian artery, and Type III involves right-sided aortic arch with isolated left subclavian artery communicating with the pulmonary artery. 3

Right aortic arch is generally an asymptomatic malformation and diagnosed incidentally. In the Type II form, in which the left subclavian artery is aberrant ( Video 1 ), patients may present to the clinic with the symptoms occurring secondary to trachea or esophagus compression or aneurysm or dissection of the vessels. 1 Dysphagia and dyspnea are usually the symptoms at the infant period, whereas atherosclerotic changes, dissection, or aneurysm may be seen in adulthood. 1 3 4

**Video 1**

Computed tomography angiography video shows right aortic arch with aberrant left subclavian artery.

Computed tomography angiography is a valuable tool for the diagnosis because of the high resolution and the speed of scanning ( Figs. 1 and 2 ). Also, magnetic resonance imaging is another option which may be used for diagnosis. 5

**Fig. 1:**
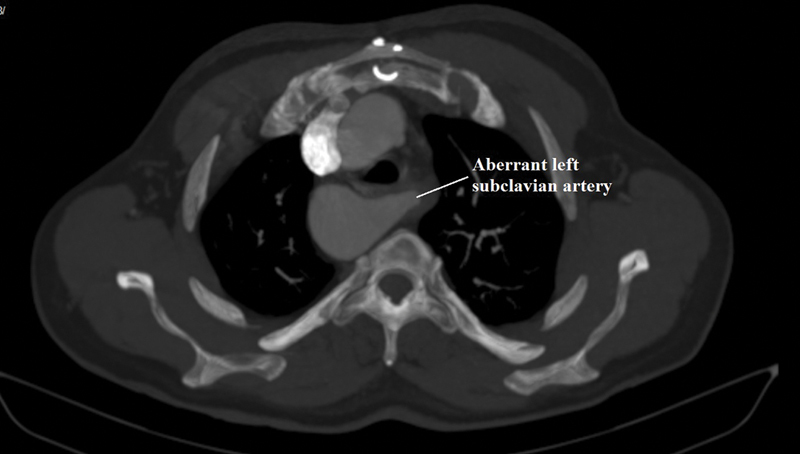
Right aortic arch with aberrant left subclavian artery.

**Fig. 2:**
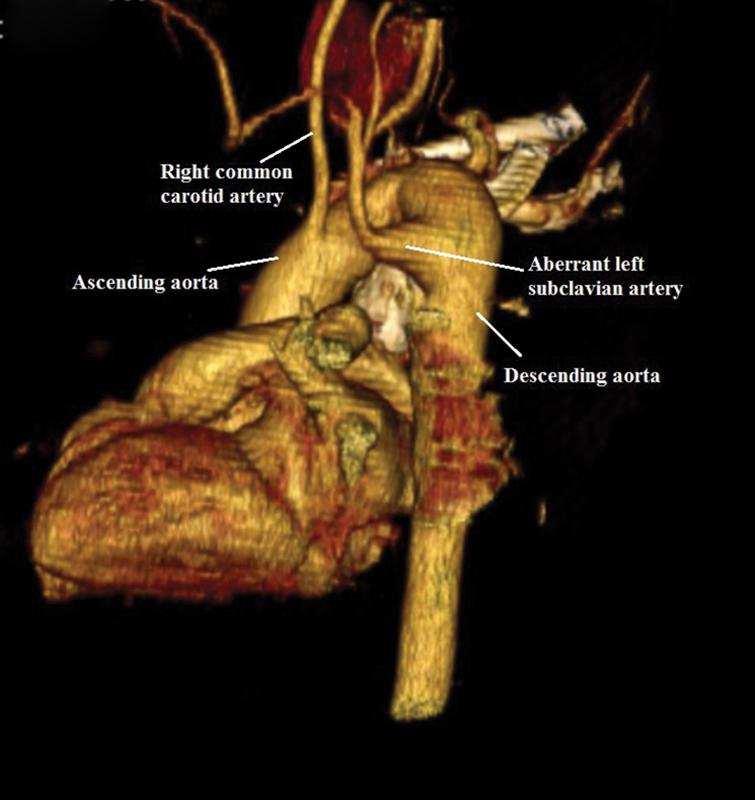
3D computed tomography angiography image. Right aortic arch with aberrant left subclavian artery. 3D, three-dimensional.

The complications of this pathology include aneurysm formation and dissection which may be secondary to atherosclerosis in latter ages, as well as recurrent lower respiratory tract infections, and growth retardation in the early years of childhood 3 ; hence, these patients should be followed-up lifelong.

In conclusion, the symptoms are the most important determinants for the treatment of the patients with RAA. Careful follow-up is necessary for the prevention of fatal complications.
